# Using Voxel-Based Morphometry to Examine the Relationship between Regional Brain Volumes and Memory Performance in Amnestic Mild Cognitive Impairment

**DOI:** 10.3389/fnbeh.2013.00089

**Published:** 2013-07-23

**Authors:** Patric Meyer, Hanna Feldkamp, Michael Hoppstädter, Andrea V. King, Lutz Frölich, Michèle Wessa, Herta Flor

**Affiliations:** ^1^Department of Cognitive and Clinical Neuroscience, Central Institute of Mental Health, Medical Faculty Mannheim, Heidelberg University, Mannheim, Germany; ^2^Division of Gerontopsychiatry, Central Institute of Mental Health, Medical Faculty Mannheim, Heidelberg University, Mannheim, Germany; ^3^Section for Experimental Psychopathology and Neuroimaging, Department of General Psychiatry, Center for Psychosocial Medicine, Heidelberg University, Heidelberg, Germany

**Keywords:** voxel-based morphometry, amnestic mild cognitive impairment, neuropsychological tests, medial temporal lobe, episodic memory, semantic memory

## Abstract

Alzheimer’s disease (AD) is a slowly progressive neurodegenerative disorder, in which morphological alterations of brain tissue develop many years before the first neuropsychological and clinical changes occur. Among the first and most prominent symptoms are deficiencies of declarative memory functions. This stage of precursory symptoms to AD has been described as amnestic mild cognitive impairment (aMCI) and is discussed as a potential AD prodrome. As therapy in the later stages of AD has been shown to be of limited impact, aMCI would be the key target for early intervention. For that purpose a comprehensive neuropsychological and anatomical characterization of this group is necessary. Previous neuropsychological investigations identified tests which are highly sensitive in diagnosing aMCI and very early AD. However, the sensitivity of those neuropsychological tests to the particular structural neuropathology in aMCI remains to be specified. To this end, we investigated 25 patients with single-domain aMCI. All participants underwent extensive neuropsychological testing and anatomical scanning with structural magnetic resonance imaging. Voxel-based morphometry (VBM) was performed to identify brain regions that show a significant correlation between regional brain volume and behavioral measures of memory and executive functioning. We found that performance in a variety of mnemonic tests was directly related to the integrity of the medial temporal lobe cortex (MTLC). Moreover, impairment of memory sub-functions in aMCI might be detected earlier than overt structural damage. By this, these findings contribute to the identification of cerebral structures associated with memory deficits in aMCI.

## Introduction

Alzheimer’s disease (AD) is a progressive neurodegenerative condition, in which neuropathological changes of brain tissue advance many years before the first clinically detectable neuropsychological alterations occur. The transitional phase in which first neuropsychological performance deficits become subjectively – and objectively – detectable but are not severe enough to be diagnosed as dementia has been labeled as mild cognitive impairment (MCI). Depending on the involvement of only one or of several cognitive domains (single-domain vs. multiple-domain MCI) and if one of the compromised functions is memory (amnestic vs. non-amnestic MCI), there are four possible subtypes of MCI (Petersen, 2004). As shown by the results of longitudinal studies, particularly the single-domain amnestic MCI (aMCI) subtype is related to an increased probability of converting to dementia whereby AD was the most prevalent demential outcome (Busse et al., 2006; Yaffe et al., 2006; Fischer et al., 2007). Hence, aMCI is rather considered to constitute a precursory phase of AD than the other subtypes. This makes a deeper investigation and understanding of aMCI-related memory impairments and their structural neural correlates a significant purpose for an early diagnosis of AD.

In the course of AD, neurofibrillary pathology, in the form of tangles made up of hyperphosphorylated tau, begins in the perirhinal cortex, and then spreads to the entorhinal cortex and the hippocampus proper (Braak and Braak, 1991). This observation is in agreement with numerous imaging studies that have found medial temporal lobe (MTL) atrophy in early AD as well as in aMCI (Pennanen et al., 2004).

Moreover, various studies have investigated the volume of the MTL with structural MRI scans in AD and MCI (aMCI or across subtypes) using manual tracing methods or voxel-based morphometry (VBM). AD patients showed significant neuronal atrophy in the MTL including the hippocampus (e.g., Karas et al., 2004; Barbeau et al., 2008; Pihlajamäki et al., 2009; Schmidt-Wilcke et al., 2009) whereas patients with MCI only displayed major neuronal loss in the medial temporal lobe cortex (MTLC) (Kordower et al., 2001).

In line with results on the contribution of myelin breakdown to AD pathology (Hyman et al., 1986; Wallin et al., 1989; Bartzokis, 2004), a further line of evidence suggests that alterations in white matter (WM) tracts which interconnect MTL structures occur before gray matter (GM) atrophy sets in and are also more severe in the preclinical phase of AD (De la Monte, 1989). Moreover, WM pathology was reported to be independent of regional cortical degeneration (Hyman et al., 1986; Delacourte et al., 1999; Kalus et al., 2006; Salat et al., 2010; Agosta et al., 2011).

Although significant neuropathological changes can be observed very early in the course of AD, it can be very difficult to diagnose aMCI since the associated cognitive deficits cannot easily be discriminated from the decline found in other cerebral disorders or in healthy aging (Petersen, 2004). However, while the memory deficits in AD are initially related to MTLC pathology, memory problems in healthy aging seem to be associated with the observed accelerated shrinkage of the hippocampus and prefrontal areas (Raz et al., 2005). In contrast, volume loss of the MTLC is minimal in healthy aging and only of a higher degree in very old age. Hence, neuropsychological tests that focus on cognitive functions relying on the integrity of the MTLC can provide an effective and sufficiently sensitive marker for an early diagnosis as those deficits should be different from deficits caused by healthy aging or other neurological or mental disorders. This may also promote early treatment allowing to slow down the progress of the disease by maintaining the patients’ present cognitive level for a longer period of time.

In order to identify those neuropsychological tests which are most sensitive in diagnosing very early AD, Swainson et al. (2001) compared the performance of patients with mild AD, aMCI, major depression, and healthy controls on a range of tests including the CANTAB (Cambridge Neuropsychological Test Automated Battery). Scores on two CANTAB tests – paired associates learning (PAL) and delayed matching to sample (DMS) as well as performance on the Wechsler Memory Scale-Revised (WMS-R; Wechsler, 1987) logical memory recall task precisely classified participants to the AD or the control group. In addition, the CANTAB PAL and DMS test scores were correlated with the degree of global cognitive decline during an 8-month follow-up period. These same two tests together with logical memory recall, semantic naming, and a category fluency (CF) task were also sensitive to deficits in aMCI when compared to the control group. Also, Égerházi et al. (2007) used the CANTAB to examine the cognitive decline of patients with aMCI and AD and confirmed that especially performance on the PAL task is a very sensitive measure to detect early neuropathological symptoms that are related to AD. In another study, Fowler et al. (2002) showed that the decline in PAL and DMS performance within a 6-month interval predicts the progression from aMCI to AD within 2 years. The WMS-R subtest logical memory, which consists of two detailed stories that subjects have to freely recall and retell, was also shown to be very sensitive to early memory impairments. Rubin et al. (1998) reported that even at a time when clinical changes were not yet evident, older subjects who later converted to AD performed significantly poorer on this test than non-converters. However, the neural origin of the sensitivity of all these neuropsychological tests for aMCI and early AD remains to be specified. Moreover, the relationship between the extent of AD-related neuropathology in aMCI and the extent of mnemonic impairment is broadly unclear up to now.

A valuable source of information on the neural basis of this sensitivity can be provided by the investigation of the relationship between inter-individual differences in performance and differences in brain structure (Kanai and Rees, 2011). In order to relate the reported neuropsychological test impairments in aMCI and early AD to underlying morphological brain changes, we used VBM on structural neuroimaging data from subjects with single-domain aMCI. As neurofibrillary pathology in AD starts in the MTLC before reaching the hippocampus, we hypothesized that performance of those neuropsychological tests from the CANTAB and the Consortium to Establish a Registry for Alzheimer’s Disease (CERAD-Plus, Morris et al., 1989) batteries which are sensitive to aMCI and early AD [Boston naming test (BNT), CF, DMS, PAL, and WMS-R logical memory recall] is mainly related to the integrity of this brain region. Only patients with aMCI were investigated in order to counteract artificially modified correlations caused by group differences in regional brain volume and memory performance.

## Materials and Methods

### Sample

Twenty-five individuals (mean age 67.96 years, SD 4.36, range 61–75, five female) who met criteria for single-domain aMCI (Petersen et al., 1999; Winblad et al., 2004) took part in the study. Participants were recruited from the Memory Clinic of the Central Institute of Mental Health. The study was approved by the ethics committee of the Medical Faculty Mannheim, University of Heidelberg and was conducted in accordance with the Declaration of Helsinki. All participants gave written informed consent prior to study start.

### Clinical assessment

All patients were diagnosed after medical and neurological examination, clinical history, scoring on the Mini-Mental State Examination (MMSE), and after neuropsychological assessment with tests assessing psychomotor speed, attention, verbal fluency, executive functions, orientation, constructional praxis, and episodic memory taken from the CERAD, the CANTAB, and the WMS-R. In addition, all participants were screened for mental disorders by the German version of the Structured Clinical Interview for DSM-IV (SCID I, Wittchen et al., 1997). All participants were German native speakers. Table 1 provides demographic characteristics and performance in neuropsychological tests. Additionally, the patient form of the Patient Competency Rating Scale (PCRS, Prigatano et al., 1986) and the PCRS relative’s form (both forms were taken from the German PCRS analog Marburger Kompetenz-Skala, MKS, Gauggel, 1998) were used as measures for ratings of impairment of daily functioning. Participants also underwent a structural MRI examination. Images were screened for probable exclusion criteria by an experienced neuroradiologist.

**Table 1:** **Demographics of the patient sample and mean scores for neuropsychological tests (standard deviation in parentheses, SD)**.

Number of subjects	25			
Mean age (years)	67.96	(4.36)		
Female/male	5/20			
Mean years of education (years)	11.28	(2.92)		
	Raw score	(SD)	Standard score*	(SD)
**ASSESSMENT OF CLINICAL/GLOBAL FUNCTIONING**				
Hamilton rating scale for depression (HAMD)	0.76	(1.27)	n/a	n/a
General depression scale (ADS-L)	7.92	(5.80)	−0.68	(0.82)
State – trait anxiety inventory – trait (STAI-T): total score	31.04	(8.25)	−0.36	(1.00)
Competence rating scale (patient form): functional abilities	102.72	(11.42)	n/a	n/a
Competence rating scale (patient form): cognitive abilities	46.56	(7.98)	n/a	n/a
Competence rating scale (relative’s form): functional abilities	107.46	(8.07)	n/a	n/a
Competence rating scale (relative’s form): cognitive abilities	50.00	(5.91)	n/a	n/a
Mehrfachwahl-Wortschatz-Intelligenztest-B (IQ-score)	101.84	(12.12)	0.16	(0.82)
CERAD: MMSE	28.16	(1.18)	−0.57	(0.99)
CANTAB simple reaction time: mean correct latency	250.36	(32.03)	n/a	n/a
**ASSESSMENT OF MNEMONIC FUNCTIONING**				
CANTAB delayed matching to sample test: percent correct (all delays)	78.93	(9.01)	0.15	(0.86)
CANTAB delayed matching to sample test: percent correct (12000ms)	70.8	(17.30)	−0.03	(1.23)
CANTAB delayed matching to sample test: percent correct (4000ms)	83.2	(11.44)	0.25	(0.78)
CANTAB paired associates learning test: total errors (6 shapes, adjusted)	9.12	(7.88)	0.06	(0.81)
CANTAB pattern recognition memory test: percent correct (immediate recall)	92.33	(9.89)	n/a	n/a
CANTAB pattern recognition memory test: percent correct (delayed recall)	74.00	(17.89)	n/a	n/a
CERAD: Boston naming test	14.28	(1.17)	0.35	(1.05)
CERAD: category fluency	20.44	(5.29)	0.29	(0.99)
Logical memory immediate (WMS-R)	31.52	(29.01)	31.52^a^	(29.01)
Logical memory delayed (WMS-R)	31.88	(29.58)	31.88^a^	(29.58)
**ASSESSMENT OF EXECUTIVE FUNCTIONING**				
CANTAB stockings of Cambridge: problems solved in minimum moves	7.6	(1.75)	−0.07	(0.90)

*Standard scores are z-values if not specified differently by a superscript letter.

*^a^The standardized mean is indicated by a percentile rank*.

*n/a Standardized scores are not available for these tests*.

*CANTAB, Cambridge Neuropsychological Test Automated Battery; CERAD, Consortium to Establish a Registry for Alzheimer’s Disease; MMSE, Mini-Mental State Examination; WMS-R, Wechsler Memory Scale-Revised*.

Participants were diagnosed as having aMCI if they fulfilled the following criteria (Petersen et al., 1999; Winblad et al., 2004): (1) concerns about memory decline, corroborated by a patient’s relative, (2) objective memory impairment defined by performance at or lower than 1.3 SDs below the mean value (i.e., under the tenth percentile) of an age- and education-matched reference population on one or more memory tests, (3) preserved general cognitive functioning defined by performance at least above 1.3 SDs below the mean on all other measures not assessing memory, (4) independence of functioning in everyday life as assessed with the PCRS, (5) not demented or suffering from conditions that also may cause memory impairments as evaluated by MRI examination, medical history, and structured clinical interviews.

All participants were required to have a negative history for neurological disorders (e.g., stroke, cerebral neoplasm, hemorrhage, inflammation, Parkinson’s disease, vascular encephalopathy with increased WM lesions), medical disorders (e.g., diabetes, untreated vitamin deficiencies, disorders of the thyroid, anemia, sleep apnea, other significant concurrent physical illnesses), and mental disorders (e.g., affective disorders). Current drug intake of dopaminergic or serotonergic agents, beta-adrenergic blockers, or benzodiazepines was ruled out. Additionally, all participants had to have normal or corrected to normal visual acuity and contrast sensitivity and had to be free of metallic biomedical implants (exclusion for MRI).

### Neuropsychological tests

#### Boston naming test

A short version of the BNT (Kaplan et al., 1983) is included in the CERAD testing battery. Participants are presented with 15 black-and-white line drawings for confrontation naming. If naming is not successful, a semantic, and if required, a phonetic cue is presented. We used the number of spontaneously correctly named items as the critical measure for the regression analyses.

#### Category fluency

In the CF task, participants have to name as many animals as possible in 1 min. The total number of items named in this interval was used in the analyses.

#### Wechsler memory scale – revised, logical memory

The WMS subtest logical memory consists of two detailed, thematically independent stories (25 items per story), that participants have to recall freely immediately after hearing and after a delay of 30 min. For analysis, we used the total number of recalled items in the immediate and in the delayed version.

#### Delayed matching to sample

The DMS assesses forced-choice recognition memory for novel visual patterns. Participants are confronted with a complex visual pattern. After a delay, this pattern is presented together with three similar patterns. The task is to identify which of these samples matches the target. The outcome measure used for regression analyses was the percentage of correct answers at the 4000 ms delay.

#### Paired associates learning

In this test, boxes are presented on the screen and opened in randomized order. A pattern is enclosed in one or more of them. Afterward, one pattern at a time is shown in the middle of the screen and the participant’s task is to indicate the box where the pattern was initially placed. In case of an error, the patterns are presented again. Test difficulty increases in terms of the number of tested patterns. We used the adjusted score of total errors at the six-pattern stage in reversed polarity for analyses. In the Swainson et al. (2001) study, this outcome measure was shown to differentiate patients with mild AD from non-demented controls with 98% accuracy.

#### Pattern recognition memory

This is a two alternatives forced-choice recognition test with abstract visual patterns. During a study phase, the participant is presented with a series of 12 visual patterns. In the recognition phase, the participant is required to choose between a pattern already seen and a new one. The percentage of correct responses was used for regression analysis.

#### Stockings of Cambridge

The stockings of Cambridge (SOC) is a spatial planning test where participants are confronted with two displays containing three colored balls. The task is to copy the pattern shown in the upper display by moving the balls in the lower display. As an expression of overall executive planning accuracy, we used the outcome measure which contains the frequency of having successfully completed a test problem in the minimum possible number of moves (Robbins et al., 1998).

### Image acquisition and analysis

Structural MRI was acquired with a 3 Tesla magnetic resonance scanner (Magnetom Trio, Siemens Medical Solutions, Erlangen, Germany). For each participant, a T1-weighted gradient echo MP-RAGE (Magnetization Prepared Rapid Gradient Echo) sequence (TR = 2300 ms, TE = 2.98 ms, flip angle 9°, FOV: 256 mm × 256 mm, voxel size: 1.0 mm × 1.0 mm × 1.1 mm, 160 sagittal slices) was acquired.

Voxel-based morphometry was applied to analyze correlations between neuropsychological test scores and GM and WM values. Contrary to manual tracing approaches, VBM permits to investigate the presence of structure-function relationships across the entire brain without *a priori* decisions about which structures to evaluate. This strategy allows to investigate if the critical tests were exclusively or especially sensitive to AD-related changes. Additionally, VBM is an automated, rater-independent method, and provides very reproducible results (Busatto et al., 2008). In contrast, the tracing procedure is dependent on unambiguous borders and anatomical landmarks and results are difficult to replicate between laboratories (Good et al., 2001). Data pre-processing and analysis were performed using SPM8^1^(Wellcome Department of Imaging Neuroscience, London, UK). Data pre-processing involved visual inspection of the T1-weighted images to control for imaging artifacts and the consecutive segmentation into GM, WM, and cerebrospinal fluid (CSF), building a customized template for GM and WM through an iteratively non-linear registration algorithm (DARTEL Toolbox for SPM8; Good et al., 2001; Ashburner, 2007) and a normalization of this template to the Montreal Neurological Institute template^2^. The Jacobian determinants resulting from the normalization procedure were used to obtain modulated VBM data preserving regional volumes. Individual GM and WM images were smoothed with an isotropic Gaussian kernel of 6 mm full-width at half-maximum before entering them into statistical analyses. Global volumes of GM, WM, and CSF were assessed from segmented images using the VBM8 toolbox for SPM8 (http://dbm.neuro.uni-jena.de/vbm8) and summed to generate an estimate for total intracranial volume (TIV). To correlate volumes and neuropsychological performance, separate test-wise regression analyses were calculated using raw scores of each test and GM and WM volumes, respectively. Age, gender, TIV as well as education were entered as covariates of no interest into all regression analyses. Results were considered significant if they consisted of at least 15 neighboring voxels that surpassed an uncorrected threshold of *p* < 0.001 (Forman et al., 1995). Given the poorer signal-to-noise ratio often observed in MTL regions due to susceptibility-related signal loss (e.g., Schacter and Wagner, 1999), a more liberal threshold of *p* < 0.005 was used for analyses within this region.

## Results

**Figure 1:**
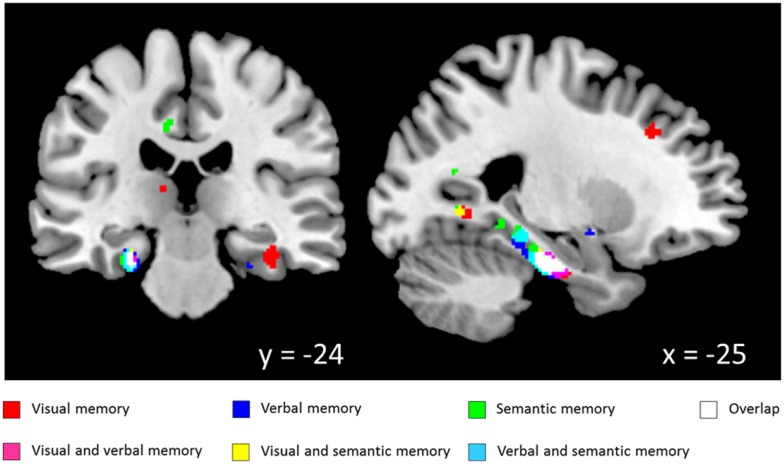
**Correlation between white matter volume and memory scores (coordinates in MNI space; thresholded at *p* < 0.005)**. *Visual memory*: paired associates learning, delayed matching to sample, pattern recognition memory; *Verbal memory*: Wechsler memory scale-revised, logical memory recall; *Semantic memory*: Boston naming test, category fluency.

White matter regression analyses demonstrated various significant positive correlations with neuropsychological test scores from the CANTAB and CERAD batteries. Significant correlations with WM volume for tests from the visual memory domain [DMS, PAL, pattern recognition memory (PRM)] were widespread but accumulated in bilateral temporal lobes including parahippocampal WM. For WMS story recall, which reflects immediate and delayed verbal memory, significant correlations were found with WM volume in the bilateral parahippocampal gyri and the left precuneus. Semantic memory test scores (CF, BNT) and WM volume were significantly correlated in the left parahippocampal gyrus. Besides task and modality specific structure-function relationships along the entire parahippocampal gyrus, a high degree of overlap of the clusters from the independent VBM analyses was found in the vicinity of the left perirhinal cortex (see Figure 1; Table 2).

**Table 2:** **Coordinates of peak value for clusters of white matter positively correlated with neuropsychological test performance**.

Test score	Region	Hemisphere	Talairach coordinates			Cluster size	Peak *T*-value
			*x*	*y*	*z*	*k*
DMS	Parahippocampal gyrus (uncus)	R	32	−4	−29	63	3.53*
	Temporal lobe (superior temporal gyrus)^a^	L	−45	−8	−7	78	4.47**
	Occipital lobe (Cuneus)^a^	L	−3	−83	25	60	4.39**
	Temporal lobe (middle temporal gyrus)	R	43	−45	6	45	3.93**
PAL	Parahippocampal gyrus	R	36	−26	−14	150	5.16**
	Frontal lobe (middle frontal gyrus)^a^	L	−38	17	25	39	4.46**
PRM	Parahippocampal gyrus	L	−23	−17	−17	87	5.11**
WMS_I	Parahippocampal gyrus	R	29	−23	−19	63	3.87*
	Parahippocampal gyrus	L	−26	−23	−21	177	5.71**
WMS_II	Temporal lobe (middle temporal gyrus)	L	−54	−7	−17	19	3.98*
	Parahippocampal gyrus	L	−23	−22	−19	119	4.56**
CF	Parahippocampal gyrus^a^	R	42	−21	−19	79	6.70**
BNT	Limbic lobe (uncus)	R	29	−4	−29	23	3.58*
	Parahippocampal gyrus	L	−24	−34	−5	45	3.46*
	Parahippocampal gyrus	L	−31	5	−30	51	6.19**
	Parahippocampal gyrus	L	−29	−18	−17	50	4.26**
	Temporal lobe (middle temporal gyrus)^a^	L	−27	−61	19	45	4.48**
	Temporal lobe (middle temporal gyrus)^a^	R	33	−55	22	24	4.26**
SOC	Frontal lobe (inferior frontal gyrus)^a^	L	−43	19	16	151	4.90**

*p < 0.005 uncorrected for multiple comparisons.

**p < 0.001 uncorrected for multiple comparisons.

*^a^For peak voxel coordinates that have been identified by the Talairach Daemon as sub-gyral, the nearest labeled gyrus/nucleus within a distance of 5 mm is given in parentheses*.

*DMS, delayed matching to sample; PAL, paired associates learning; PRM, pattern recognition memory; WMS_I, Wechsler Memory Scale-Revised, logical memory immediate recall; WMS_II, Wechsler Memory Scale-Revised, logical memory delayed recall; CF, category fluency; BNT, Boston naming test; SOC, Stockings of Cambridge*.

In contrast to the memory-dependent measures, performance scores of the SOC, reflecting executive functioning, revealed significant correlations with WM values localized in the left frontal lobe.

No coherent results were obtained in the regression analysis with regard to GM volumes.

## Discussion

The present study revealed the neural correlates of different cognitive sub-functions assessed by neuropsychological tests that are most commonly used for diagnosing aMCI and early AD. VBM analyses demonstrated that performance in a variety of mnemonic tests is directly related to the integrity of the MTLC in patients with single-domain aMCI. In more detail, performance in BNT, CF, PAL, DMS, PRM as well as WMS was mainly associated with reduced WM volume in the perirhinal and entorhinal region. Although no exact tract definition is possible using VBM-derived data, it is likely that the perforant path, which provides the major input route to the hippocampus as well as the cingulum bundle connecting the various components of the limbic system, are included in the reported MTLC WM clusters. In contrast, a significant association of measures of executive functioning and WM volume was found in the frontal lobe. Retrospectively, these tests were found to be sensitive to MCI and early AD in previous studies because they rely on the integrity of those brain structures which are specifically injured in these conditions. These findings are consistent with the specific pathology in the AD process (Hyman et al., 1986; Wallin et al., 1989; Bartzokis, 2004) and support the notion of a very early involvement of the MTLC during the disease process. Previous comparisons of MCI patients to healthy controls showed different forms of brain atrophy depending on the nature and state of the cognitive impairment (e.g., Whitwell et al., 2007). Interestingly, a cohort analysis revealed no decreases in GM and WM volumes in the MTL in our patients with aMCI. This might be due to the fact that they are at a very early stage of the disease process so that a subtle decline in regional GM and WM volume is not detectable yet, at least not when using VBM. Nevertheless, even in the absence of visible atrophy on MRI scans, the result of the regression analyses demonstrated a clear relationship between the mnemonic impairments and the integrity of the MTLC. This fits well to the results of a recent meta-analysis by Schmand et al. (2010) who concluded that memory impairment is a much more precise early predictor of AD than local brain atrophy. Recently, Leal and Yassa (2013) argued that the sensitivity of AD markers may vary as a function of how far patients are in the disease process with small changes in the perforant path being a much more salient feature than frank volume loss in the MTL in the early MCI phase. The authors concluded that in the absence of overt structural decline, functional markers are most important.

Contrary to our expectations, we could not observe a structure-function relationship with regard to GM. Similarly to the missing signs of clear MTLC atrophy in our patient sample, this might be caused by the very early state of the cognitive impairment. As mentioned above, alterations in WM tracts connecting MTL structures seem to occur before cortical degeneration starts and were also found to be partly independent of it (Delacourte et al., 1999; Kalus et al., 2006; Salat et al., 2010). In a postmortem investigation of very mild AD patients, Hyman et al. (1986) could demonstrate that cell loss in the MTLC causes a disconnection of the hippocampus from cortical inputs and that this disconnection was related to memory impairments. Recent diffusion tensor imaging and VBM studies confirmed that it is the MTLC WM that is affected most prominently in aMCI (Salat et al., 2010) and AD (Stoub et al., 2006) and that those alterations are related to later memory impairments (Salat et al., 2010). In addition, Zhuang et al. (2012) investigated retrospectively whether microstructural WM changes are already present in cognitively normal individuals without dementia who will later develop aMCI. At baseline, converters compared to non-converters showed substantial reductions in WM integrity in the MTLC whose degree was correlated with greater verbal episodic memory decline. In contrast, GM density was found not to be related to longitudinal episodic memory loss.

A considerable number of findings indicate that the involvement of the MTLC in binding seems both necessary and sufficient for the formation of semantic representations, i.e., semantic memories of events (e.g., Taylor et al., 2006). By binding associative or contextual information together with the semantic item representation, the hippocampus instead provides both necessary and sufficient conditions for the formation of episodic memories (e.g., Aggleton and Brown, 2006). However, due to this probable interruption of information flow between the different MTL structures, MTLC and hippocampus were not differently associated with semantic and episodic memory processes in our study. Although recall and cued recall tests normally rely more on the hippocampus (Golomb et al., 1994; Pohlack et al., 2013), performance in the WMS logical memory recall task or in the PAL were related to MTLC integrity in our sample. However, WM volume of different MTLC areas was differently associated with certain memory sub-functions with regard to laterality. Both PAL and DMS performance primarily correlated with WM volume in the area of the right parahippocampal gyrus while semantic memory as measured by CF and BNT was associated with left hemisphere WM volume.

Within this context, it is important to note that we did not identify the brain regions underlying the specific task-relevant cognitive processes *per se* as only participants in a pathological condition were investigated. Consequently, it is very likely that due to the probable disconnection of MTL structures, reorganization, or compensation processes are at work in aMCI patients. This precludes any clear inferences to intact cognitive processes in a normal healthy population. Here, we asked instead why specific neuropsychological tests have previously been found to be sensitive for aMCI and early AD.

Besides methodological differences, the position of our patients on a very early stage of the disease process may also account for different findings in the literature relating to the reported regression analyses (e.g., Schmidt-Wilcke et al., 2009). Chételat et al. (2003) found that deficits in both word list encoding and retrieval, were correlated with a decline in hippocampal GM in patients with single-domain aMCI. In contrast to our study, only GM density in patients diagnosed using a more conservative performance cut-off score (1.5 SDs instead of 1.3 SDs as in the present study) was used. This was also the case in a study by Barbeau et al. (2008) who reported that in aMCI patients with preserved recognition and impaired recall, GM density was reduced in frontal areas while aMCI patients with impaired recall and recognition memory showed reduced density in the right MTL and bilateral temporo-parietal regions. In addition, the diagnostic sensitivity of the tests applied in these investigations is so far unclear. A cohort analysis conducted by Schmidt-Wilcke et al. (2009) revealed bilateral decreases in GM density in the MTLs and the lateral temporal lobes when aMCI patients were compared to healthy controls. Additional regression analyses showed a significant positive association between immediate verbal recall and GM integrity in the left MTLC while delayed free recall correlated with GM density in the hippocampus proper. However, in this study also patients with multiple-domain aMCI were enrolled. This was also the case in a recent study by Barbeau et al. (2012) which demonstrated significant positive correlations between semantic memory scores and MTLC as well as anterior hippocampus GM density. However, as the kind of conversion of patients suffering from multiple-domain aMCI is less clear (Petersen, 2004; Fischer et al., 2007), these data are not directly comparable to the results of the present study that only incorporated patients with single-domain aMCI, who are probably located at a transitional stage toward the development of AD. Thus, the current results obtained in a more homogenous sample at a probable earlier time point in the disease process argue even more strongly in favor of the diagnostic value of the reported memory tests.

## Conclusion

In a homogenous sample of patients with single-domain aMCI, we detected relationships between brain structure and mnemonic function in regions closely similar to previously established early morphological alterations in aMCI and AD even before those structural manifestations are detectable. By this, the present results suggest that performance in frequently used neuropsychological memory tests can predict the integrity of the cortical regions which are affected first by AD pathology such as the perirhinal cortex.

However, it should be kept in mind that this study also has some limitations. Due to the exploratory purpose of this study and the poorer signal-to-noise ratio often observed in MTL regions due to susceptibility-related signal loss, a more liberal threshold of *p* < 0.005 together with a cluster-threshold of at least 15 neighboring voxels was used for analyses within this region. When using FWE as correction, none of the results survived. Moreover, the present sample was diagnosed with the same tests that were used for regression analyses. In addition no control group was tested. Consequently, the observed correlations are not necessarily aMCI-specific. Nevertheless, they demonstrate why specific neuropsychological tests have previously been found to be sensitive for aMCI and early AD.

## Conflict of Interest Statement

The authors declare that the research was conducted in the absence of any commercial or financial relationships that could be construed as a potential conflict of interest.

## Footnotes

^1^http://www.fil.ion.ucl.ac.uk/spm/software/spm8

^2^http://www.loni.ucla.edu/ICBM/ICBM_Databases.html

## References

[B1] Aggleton J. P. Brown M. W. (2006). Interleaving brain systems for episodic and recognition memory. Trends Cogn. Sci. (Regul. Ed.) 10, 455–463 10.1016/j.tics.2006.08.003 16935547

[B2] Agosta F. Pievani M. Sala S. Geroldi C. Galluzzi S. Frisoni G. B. (2011). White matter damage in Alzheimer disease and its relationship to gray matter atrophy. Radiology 258, 853–863 10.1148/radiol.10101284 21177393

[B3] Ashburner J. (2007). A fast diffeomorphic image registration algorithm. Neuroimage 38, 95–113 10.1016/j.neuroimage.2007.07.007 17761438

[B4] Barbeau E. Didic M. Joubert S. Guedj E. Koric L. Felician O. R. (2012). Extent and neural basis of semantic memory impairment in mild cognitive impairment. J. Alzheimers Dis. 28, 823–837 10.3233/JAD-2011-110989 22112550

[B5] Barbeau E. J. Ranjeva J. P. Didic M. Confort-Gouny S. Felician O. Soulier E. (2008). Profile of memory impairment and gray matter loss in amnestic mild cognitive impairment. Neuropsychologia 46, 1009–1019 10.1016/j.neuropsychologia.2007.11.019 18191160

[B6] Bartzokis G. (2004). Age-related myelin breakdown: a developmental model of cognitive decline and Alzheimer’s disease. Neurobiol. Aging 25, 5–18 10.1016/j.neurobiolaging.2003.03.001 14675724

[B7] Braak H. Braak E. (1991). Neuropathological stageing of Alzheimer-related changes. Acta Neuropathol. 82, 239–259 10.1007/BF00308809 1759558

[B8] Busatto G. F. Diniz B. S. Zanetti M. V. (2008). Voxel-based morphometry in Alzheimer’s disease. Expert Rev. Neurother. 8, 1691–1702 10.1586/14737175.8.11.1691 18986240

[B9] Busse A. Hensel A. Gühne U. Angermeyer M. C. Riedel-Heller S. G. (2006). Mild cognitive impairment: long-term course of four clinical subtypes. Neurology 67, 2176–2185 10.1212/01.wnl.0000249117.23318.e1 17190940

[B10] Chételat G. Desgranges B. de la Sayette V. Viader F. Berkouk K. Landeau B. (2003). Dissociating atrophy and hypometabolism impact on episodic memory in mild cognitive impairment. Brain 126, 1955–1967 10.1093/brain/awg196 12821520

[B11] De la Monte S. M. (1989). Quantitation of cerebral atrophy in preclinical and end-stage Alzheimer’s disease. Ann. Neurol. 25, 450–459 10.1002/ana.410250506 2774485

[B12] Delacourte A. David J. P. Sergeant N. Buée L. Wattez A. Vermersch P. (1999). The biochemical pathway of neurofibrillary degeneration in aging and Alzheimer’s disease. Neurology 52, 1158–1165 10.1212/WNL.52.6.1158 10214737

[B13] Égerházi A. Berecz R. Bartók E. Degrell I. (2007). Automated neuropsychological test battery (CANTAB) in mild cognitive impairment and in Alzheimer’s disease. Prog. Neuropsychopharmacol. Biol. Psychiatry 31, 746–751 10.1016/j.pnpbp.2007.01.011 17289240

[B14] Fischer P. Jungwirth S. Zehetmayer S. Weissgram S. Hoenigschnabl S. Gelpi E. (2007). Conversion from subtypes of mild cognitive impairment to Alzheimer dementia. Neurology 68, 288–291 10.1212/01.wnl.0000252358.03285.9d 17242334

[B15] Forman S. D. Cohen J. D. Fitzgerald M. Eddy W. F. Mintun M. A. Noll D. C. (1995). Improved assessment of significant activation in functional magnetic resonance imaging fMRI: use of a cluster-size threshold. Magn. Reson. Med. Sci. 33, 636–647 10.1002/mrm.1910330508 7596267

[B16] Fowler K. S. Saling M. M. Conway E. L. Semple J. M. Louis W. J. (2002). Paired associate performance in the early detection of DAT. J. Int. Neuropsychol. Soc. 8, 58–71 10.1017/S1355617702811067 11843075

[B17] Gauggel S. (ed.) (1998). MarburgerKompetenz-Skala (MKS). Marburg: University of Marburg

[B18] Golomb J. Kluger A. de Leon M. J. Ferris S. H. Convit A. Mittelman M. S. (1994). Hippocampal formation size in normal human aging: a correlate of delayed secondary memory performance. Learn. Mem. 1, 45–54 10467585

[B19] Good C. D. Johnsrude I. S. Ashburner J. Henson R. N. A. Friston K. J. Frackowiak R. S. J. (2001). A voxel-based morphometric study of ageing in 465 normal adult human brains. Neuroimage 14, 21–36 10.1006/nimg.2001.0857 11525331

[B20] Hyman B. T. Van Hoesen G. W. Kromer L. J. Damasio A. R. (1986). Perforant pathway changes and the memory impairment of Alzheimer’s disease. Ann. Neurol. 20, 472–481 10.1002/ana.410200406 3789663

[B21] Kalus P. Slotboom J. Gallinat J. Mahlberg R. Cattapan-Ludewig K. Wiest R. (2006). Examining the gateway to the limbic system with diffusion tensor imaging: the perforant pathway in dementia. Neuroimage 30, 713–720 10.1016/j.neuroimage.2005.10.035 16337815

[B22] Kanai R. Rees G. (2011). The structural basis of inter-individual differences in human behaviour and cognition. Nat. Rev. Neurosci. 12, 231–242 10.1038/nrn3000 21407245

[B23] Kaplan E. Goodglass H. Weintraub S. Segal O. (1983). Boston Naming Test. Philadelphia: Lea & Febiger

[B24] Karas G. B. Scheltens P. Rombouts S. A. Visser P. J. van Schijndel R. A. Fox N. C. (2004). Global and local gray matter loss in mild cognitive impairment and Alzheimer’s disease. Neuroimage 23, 708–716 10.1016/j.neuroimage.2004.07.006 15488420

[B25] Kordower J. H. Chu Y. Stebbins G. T. DeKosky S. T. Cochran E. J. Bennett D. (2001). Loss and atrophy of layer II entorhinal cortex neurons in elderly people with mild cognitive impairment. Ann. Neurol. 49, 202–213 10.1002/1531-8249(20010201)49:2<202::AID-ANA40>3.0.CO;2-3 11220740

[B26] Leal S. L. Yassa M. A. (2013). Perturbations of neural circuitry in aging, mild cognitive impairment, and Alzheimer’s disease. Ageing Res. Rev. 10.1016/j.arr.2013.01.006 23380151 PMC3893046

[B27] Morris J. C. Heyman A. Mohs R. C. Hughes J. P. van Belle G. Fillenbaum G. (1989). The Consortium to Establish a Registry for Alzheimer’s Disease CERAD Part I clinical and neuropsychological assessment of Alzheimer’s disease. Neurology 39, 1159–1165 10.1212/WNL.39.9.1159 2771064

[B28] Pennanen C. Kivipelto M. Tuomainen S. Hartikainen P. Hänninen T. Laakso M. P. (2004). Hippocampus and entorhinal cortex in mild cognitive impairment and early AD. Neurobiol. Aging 25, 303–310 10.1016/S0197-4580(03)00084-8 15123335

[B29] Petersen R. C. (2004). Mild cognitive impairment as a diagnostic entity. J. Intern. Med. 256, 183–194 10.1111/j.1365-2796.2004.01388.x 15324362

[B30] Petersen R. C. Smith G. E. Waring S. C. Ivnik R. J. Tangalos E. G. Kokmen E. (1999). Mild cognitive impairment: clinical characterization and outcome. Arch. Neurol. 56, 303–308 10.1001/archneur.56.3.303 10190820

[B31] Pihlajamäki M. Jauhiainen A. M. Soininen H. (2009). Structural and functional MRI in mild cognitive impairment. Curr. Alzheimer Res. 6, 179–185 10.2174/156720509787602898 19355853

[B32] Pohlack S. T. Meyer P. Cacciaglia R. Liebscher C. Ridder S. Flor H. (2013). Bigger is better! Hippocampal volume and declarative memory performance in healthy young men. Brain Struct. Funct. 10.1007/s00429-012-0497-z 23269366 PMC3889822

[B33] Prigatano G. P. Fordyce D. J. Zeiner H. K. Roueche J. R. Pepping M. Wood B. C. (1986). Neuropsychological Rehabilitation after Brain Injury. Baltimore: John Hopkins University Press

[B34] Raz N. Lindenberger U. Rodrigue K. M. Kennedy K. M. Head D. Williamson A. (2005). Regional brain changes in aging healthy adults: general trends, individual differences and modifiers. Cereb. Cortex 15, 1676–1689 10.1093/cercor/bhi044 15703252

[B35] Robbins T. W. James M. Owen A. M. Sahakian B. J. Lawrence A. D. McInnes L. (1998). A study of performance on tests from the CANTAB battery sensitive to frontal lobe dysfunction in a large sample of normal volunteers: implications for theories of executive functioning and cognitive aging. Cambridge Neuropsychological Test Automated Battery. J. Int. Neuropsychol. Soc. 4, 474–490 9745237 10.1017/s1355617798455073

[B36] Rubin E. H. Storandt M. Miller J. P. Kinscherf D. A. Grant E. A. Morris J. C. (1998). A prospective study of cognitive function and onset of dementia in cognitively healthy elders. Arch. Neurol. 55, 395–401 10.1001/archneur.55.3.395 9520014

[B37] Salat D. H. Tuch D. S. van der Kouwe A. J. Greve D. N. Pappu V. Lee S. Y. (2010). White matter pathology isolates the hippocampal formation in Alzheimer’s disease. Neurobiol. Aging 31, 244–256 10.1016/j.neurobiolaging.2008.03.013 18455835 PMC3038572

[B38] Schacter D. L. Wagner A. D. (1999). Medial temporal lobe activations in fMRI and PET studies of episodic encoding and retrieval. Hippocampus 9, 7–24 10.1002/(SICI)1098-1063(1999)9:1<7::AID-HIPO2>3.0.CO;2-K 10088896

[B39] Schmand B. Huizenga H. M. van Gool W. A. (2010). Meta-analysis of CSF and MRI biomarkers for detecting preclinical Alzheimer’s disease. Psychol. Med. 40, 135–145 10.1017/S0033291709991516 19863841

[B40] Schmidt-Wilcke T. Poljansky S. Hierlmeier S. Hausner J. Ibach B. (2009). Memory perdormance correlates with gray matter density in the ento-/perirhinal cortex and posterior hippocampus in patients with mild cognitive impairment and healthy controls – a voxel based morphometry study. Neuroimage 47, 1914–1920 10.1016/j.neuroimage.2009.04.092 19442751

[B41] Stoub T. R. de Toledo-Morrell L. Stebbins G. T. Leurgans S. Bennett D. A. Shah R. C. (2006). Hippocampal disconnection contributes to memory dysfunction in individuals at risk for Alzheimer’s disease. Proc. Natl. Acad. Sci. U.S.A. 103, 10041–10045 10.1073/pnas.0603414103 16785436 PMC1479867

[B42] Swainson R. Hodges J. R. Galton C. J. Semple J. Michael A. Dunn B. D. (2001). Early detection and differential diagnosis of Alzheimer’s disease and depression with neuropsychological tasks. Dement. Geriatr. Cogn. Disord. 12, 265–280 10.1159/000051269 11351138

[B43] Taylor K. I. Moss H. E. Stamatakis E. A. Tyler L. K. (2006). Binding crossmodal object features in perirhinal cortex. Proc. Natl. Acad. Sci. U.S.A. 103, 8239–8244 10.1073/pnas.0509704103 16702554 PMC1461402

[B44] Wallin A. Gottfries C. G. Karlsson I. Svennerholm L. (1989). Decreased myelin lipids in Alzheimer’s disease and vascular dementia. Acta Neurol. Scand. 80, 319–323 10.1111/j.1600-0404.1989.tb03886.x 2816288

[B45] Wechsler D. (1987). Wechsler Memory Scale-Revised. New York: Harcourt Brace Jovanovich

[B46] Whitwell J. L. Petersen R. C. Negash S. Weigand S. D. Kantarci K. Ivnik R. J. (2007). Patterns of atrophy differ among specific subtypes of mild cognitive impairment. Arch. Neurol. 64, 1130–1138 10.1001/archneur.64.8.1130 17698703 PMC2735186

[B47] Winblad B. Palmer K. Kivipelto M. Jelic V. Fratiglioni L. Wahlund L. O. (2004). Mild cognitive impairment – beyond controversies, towards a consensus: report of the International Working Group on Mild Cognitive Impairment. J. Intern. Med. 256, 240–246 10.1111/j.1365-2796.2004.01380.x 15324367

[B48] Wittchen H. U. Wunderlich U. Gruschwitz S. Zaudig M. (1997). SKID-I. Strukturiertes Klinisches Interview für DSM-IV. Göttingen: Hogrefe

[B49] Yaffe K. Petersen R. C. Lindquist K. Kramer J. Miller B. (2006). Subtype of mild cognitive impairment and progression to dementia and death. Dement. Geriatr. Cogn. Disord. 22, 312–319 10.1159/000095427 16940725

[B50] Zhuang L. Sachdev P. S. Trollor J. N. Kochan N. A. Reppermund S. Brodaty H. (2012). Microstructural white matter changes in cognitively normal individuals at risk of amnestic MCI. Neurology 79, 748–754 10.1212/WNL.0b013e3182661f4d 22843270

